# Neuro-Immune Mechanisms of Anti-Cryptococcal Protection

**DOI:** 10.3390/jof4010004

**Published:** 2017-12-25

**Authors:** Rebecca A. Drummond

**Affiliations:** Fungal Pathogenesis Unit, Laboratory of Clinical Immunology and Microbiology, National Institutes of Health, Bethesda, MD 20892, USA; rebecca.drummond@nih.gov; Tel.: +1-301-761-6622

**Keywords:** *Cryptococcus*, microglia, astrocytes, copper, meningitis, neuron, antifungal immunity

## Abstract

Cryptococcal meningitis (CM) is a life-threatening fungal disease affecting both immunosuppressed and immunocompetent people. The main causative agent of CM is *Cryptococcus neoformans*, a basidiomycete fungus prevalent in the environment. Our understanding of the immune mechanisms controlling *C. neoformans* growth within the central nervous system (CNS) is poor. However, there have been several recent advances in the field of neuroimmunology regarding how cells resident within the CNS, such as microglia and neurons, can participate in immune surveillance and control of infection. In this mini-review, the cells of the CNS are discussed with reference to what is currently known about how they control *C. neoformans* infection.

## 1. Introduction

The central nervous system (CNS) is prone to infection from a wide range of micro-organisms including bacteria, viruses, parasites, and fungi. Many CNS-resident cells are now understood to participate in immune responses, including microglia and astrocytes, which has led to a growing appreciation of the dynamic immune kinetics within the CNS and a greater understanding that this organ is not merely a bystander in preventing infection. However, many of the immune mechanisms controlling CNS infection are only partially understood and this is particularly true of CNS infections caused by fungi. 

The most common causative agent of fungal meningitis is the *Cryptococcus* species, particularly *C. neoformans* and *C. gattii*. These are basidiomycete fungi prevalent in the environment that are inhaled into the lungs, where they are either rapidly killed by lung-resident phagocytes or potentially establish latent infections [1]. Should these killing and/or control mechanisms fail, yeast cells in the lung may germinate, resulting in pulmonary infection and subsequent dissemination to the CNS, where they cause cryptococcal meningitis (CM), a serious disease associated with high mortality and lasting neurological defects in survivors [2]. Development of CM is most strongly associated with prior HIV infection, indicating that proper induction of CD4^+^ T-cell responses is critical [3]. Further risk factors for CM include the use of immunosuppressants, idiopathic CD4 T-cell lymphocytopenia, and circulating autoantibodies [4], although we do not yet have a complete understanding of the full range of risk factors that enhance susceptibility to cryptococcal diseases.

In the lungs, control of *C. neoformans* infections depends on the polarization of CD4^+^ T-cells towards the IFNγ producing T-helper 1 (Th1) phenotype and recruitment of monocyte-derived dendritic cells (DCs) [5]. Th1 cells in turn help drive “classic activation” of macrophages (M1), which express enhanced levels of inducible nitric oxide synthase (iNOS) that are important for fungal killing [5]. These protective immune mechanisms can be counteracted by *C. neoformans*’ intricate survival systems [6], including the ability to actively block maturation of phagosomes by catalyzing the removal of Rab guanosine triphosphate hydrolases (GTPases) to help neutralize acidification of the phagosome [7], and through lysosome damage [8]. Moreover, *C. neoformans* has also been shown to exit macrophages following phagocytosis using a non-lytic extrusion mechanism (termed vomocytosis) that is prevented by actin polymerization [9] and ERK5 signaling in macrophages [10]. In the brain, our understanding of the host–pathogen interactions that occur are much less clear, in part because we are only beginning to understand the control of CNS immune responses. This brief review discusses the major cellular components of the CNS and summarizes what we currently know about how they work to prevent *C. neoformans* infection and the development of CM.

## 2. Microglia

Microglia are the resident macrophages of the CNS with the predominant function of immune surveillance, and as a result the majority of studies analyzing immunity to CNS infections focus on these cells. Microglia are found throughout the brain parenchyma in a relatively homogenous fashion, forming long dendrites that probe their surrounding environment [11]. Unlike most immune cells in the body, microglia do not generate from the bone marrow and instead develop from yolk-sac precursors that seed the developing brain during embryogenesis [12], self-renewing throughout life to form clonal populations [13] that range in age from months to years old [14].

Since microglia are related to macrophages, they are equipped with the necessary immune arsenal to deal with invading fungi. Microglia express pattern recognition receptors (PRRs) including the Toll-like receptors (TLRs) and C-type lectin receptors (CLRs), which recognize fungal pathogen-associated molecular patterns (PAMPs) [15]. Using genetically-deficient mice, recognition of *C. neoformans* and restriction of its dissemination to the CNS seems to depend predominantly on the TLRs [16], whereas “classic” anti-fungal CLRs, such as Dectin-1, have no role [17]. This is likely because *C. neoformans* forms a large polysaccharide capsule, which masks available PAMPs in the yeast cell wall, resulting in limited recognition of *C. neoformans* yeast by many of the CLRs [17], although recognition of infectious spores in the lung by alveolar macrophages does require Dectin-1 [18]. The exact roles of these innate receptors specifically expressed by microglia, their functions in preventing development of CM, and whether the spore form can even disseminate to the CNS are not well understood.

**Figure 1:**
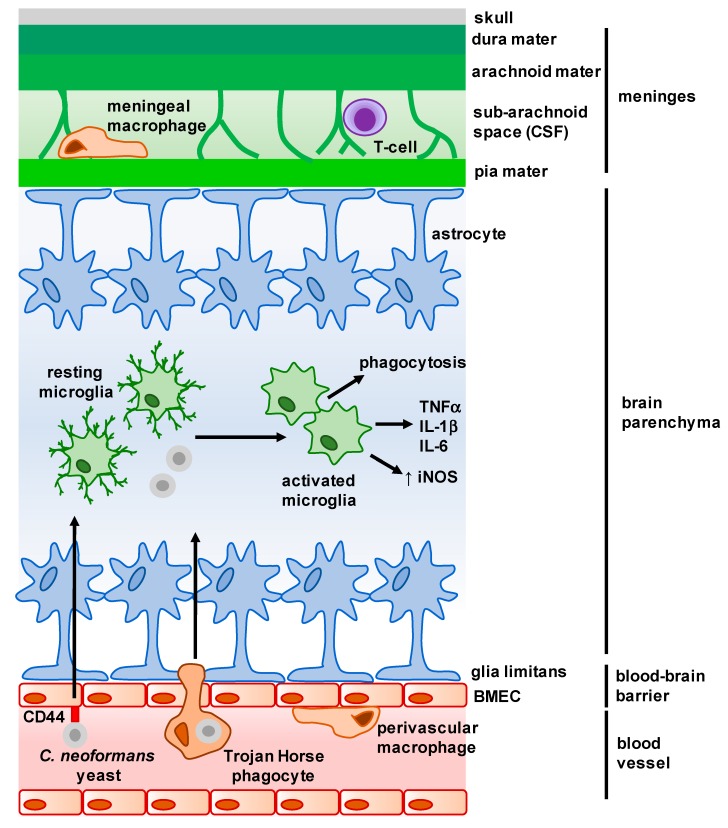
Overview of the main structures and cells of the central nervous system (CNS). The meninges is a complex membrane structure surrounding the brain and spinal cord, and is composed of a series of layers in which several immune cells are found. Cerebrospinal fluid (CSF) flows through the sub-arachnoid space here, and drains into the lymph nodes via lymphatic vessels (not shown). Astrocytes form barriers along the meninges and blood–brain barrier (BBB) and largely control cellular movement into the CNS. Microglia are found within the parenchyma, and exhibit a dendritic morphology when resting. Upon interaction with microbes, microglia activate and appear amoeboid in morphology. *C. neoformans* enters the parenchyma either as free yeast cells (following interaction with brain microvascular endothelial cells (BMECs)) or within infected macrophages (Trojan Horse phagocytes) from the brain microvasculature. Arrows represent movement of *C. neoformans* into the brain parenchyma, and resulting activation and cytokine production by microglia.

Intracellular signaling cascades initiated by microglia-expressed PRRs following PAMP binding activate multiple antifungal responses to limit fungal growth (Figure 1). For example, microglia produce multiple pro-inflammatory cytokines upon *C. neoformans* exposure including TNFα, IL-1β and IL-6 [19] and upregulate activation markers such as MHC Class II and CD11c [20]. Moreover, microglia are also able to phagocytose *C. neoformans* yeast cells and upregulate expression of iNOS for fungal killing [21,22], although this appears to be largely dependent on prior opsonization of yeast cells and microglia expression of the receptor GPR43 [23,24]. Despite the phagocytic capabilities of microglia, they do not appear to be able to kill yeast cells efficiently [24] and are susceptible to latent intracellular infection [25].

In addition to their roles in innate immunity, microglia are also able to participate in the induction of adaptive immune responses [15]. Disturbances in CD4^+^ T-cell function most closely associate with the development of CM in humans, and thus the appropriate activation of T-cell responses is critical for containing infection. Using in vitro assays, microglia were shown to express MHC Class II and interact with cryptococcal-specific CD4^+^ T-cells [26]. In contrast, microglia did not efficiently interact with CD8^+^ T-cells [26], in line with the observation that control of brain infection depends heavily on CD4^+^ T-cells and less so on CD8^+^ T-cells, while pulmonary containment relies on both lymphocyte subsets [27]. It is interesting to note, however, that microglia may not be the only, or even the predominant, antigen-presenting cells (APCs) in the brain. DCs are the prototypal APC, with resident organ-specific populations found in nearly every tissue. It was long thought that the CNS was devoid of DCs; however, they have been shown to accumulate in the CNS during parasitic and bacterial meningitis [28], and increased numbers of myeloid DCs have been reported in the cerebrospinal fluid (CSF) of patients with severe CM [29]. The function of these infiltrating DCs is not yet clear; further study into their kinetics and possible protective capacities may yield interesting new insights into the pathogenesis of infectious meningitis in the future.

## 3. Non-Parenchymal Macrophages

In addition to microglia, there are populations of non-parenchymal macrophages (NPMs) found in the brain that are poorly understood, partly because there is a significant overlap of surface marker expression between NPMs and microglia and a paucity of specific markers for the different subsets [30]. NPMs include perivascular macrophages, choroid plexus macrophages, and meningeal macrophages and are reviewed extensively elsewhere [31], but summarized briefly in Table 1.

Microscopic analysis of human brain tissue has shown that perivascular macrophages phagocytose *C. neoformans* [32] and thus may be involved in the initial control of brain infection as disseminated yeast cells interact with and invade the CNS vasculature. Indeed, early studies concluded that perivascular macrophages, and not microglia, were the key cell type mediating fungal resistance in the brain [33]. However, these early conclusions were based on assumptions that microglia and perivascular macrophages had different origins, but we now know that they share embryonic beginnings and cannot be distinguished based on their turnover rates [31]. The only resident macrophage population in the brain that has been shown to have at least partial turnover by circulating monocytes is choroid plexus macrophages. The choroid plexus makes CSF, and therefore these macrophages are thought to play key roles in the recognition and clearance of antigen from CSF [31]. *C. neoformans* infections of the choroid plexus are rare [34], possibly because *C. neoformans* is not thought to cross into the brain via this structure (discussed below). It is therefore unlikely that this NPM population is involved in anti-cryptococcal immunity; however, their role, along with the other NPM populations, is largely unexplored in the cryptococcal field.

## 4. Macroglia: Astrocytes and Oligodendrocytes

The most numerous cells in the CNS are the macroglia, which include the astrocytes and oligodendrocytes. Macroglia perform many functions but are key for the homeostatic functioning of the CNS through the provision of structural and nutritional support for neurons and endothelial cells [35].

Astrocytes are a major component of the blood–brain barrier (BBB). Borders of endothelial cells are joined by tight junctions and occludin proteins and form the “primary” BBB, however beyond this there is a complex network of astrocyte processes termed “end-feet”, which are tightly bound together and form the secondary component of the BBB called the glia limitans [36] (Figure 1). Astrocytes in the glia limitans regulate the trafficking of leukocytes across the BBB, by either restricting movement through the formation of tight junctions [36] or promoting leukocyte accumulation via the production of chemokines [35]. Further to their role as gatekeepers of the CNS parenchyma, astrocytes have also been shown to be directly involved in CNS immune responses. During infection or injury, astrocytes undergo a complex, poorly-understood process termed ‘astrogliosis,’ which involves significant structural and functional changes. These changes are directly regulated by the microenvironment, giving rise to distinct functional phenotypes that are either optimized to promote resistance to infection (through the expression of complement proteins) or mediating tissue repair (through the expression of neurotrophic factors), and this is often driven by the activation status of microglia [37,38].

There is very little understood about how astrocytes might participate in immunity to *C. neoformans* and their role in CM pathogenesis. Astrogliosis has been reported in animals infected with *C. neoformans* [39], and studies using in vitro models demonstrated that astrocytes upregulate MHC Class II expression following *C. neoformans* stimulation [40], suggesting that these cells actively respond to infection and could potentially activate protective T-cell responses. However, astrogliosis is not always protective in the context of infection. In bacterial meningitis, highly reactive astrocytes are associated with immunopathology within the CNS and targeting these pathways can help alleviate meningitis symptoms and promote recovery [41].

Oligodendrocytes are susceptible to viral infection and have not been reported to have particularly heavy involvement during CNS infection with other pathogens. Oligodendrocytes have, however, been shown to secrete complement proteins when stimulated with *Aspergillus fumigatus*, a common fungal pathogen in immunosuppressed humans [42]. Whether similar reactions occur during CM, and if *C. neoformans* infects oligodendrocytes, is not known.

## 5. Neurons

While not typically considered for their role in immunity, recent studies have demonstrated that neurons, particularly sensory neurons, can function to alert surrounding tissues to infection or damage and directly sense microbes [43]. Whether neurons in the brain become damaged by *C. neoformans* infection or are able to sense and respond to this damage is not known; however, it is interesting to note that strict control of copper homeostasis is needed to maintain neuronal health [44], which may impact on *C. neoformans* pathogenesis within the brain.

Copper is an essential trace element for all living organisms, acting as a critical enzyme co-factor and is involved in other cellular processes such as managing intracellular iron levels [45]. However, high copper concentrations are toxic and contribute towards pathologies, particularly in the CNS [44]. Neurons express high levels of copper transporters, such as Ctr1 and ATP7A, and these expression patterns vary depending on anatomical location within the brain [46]. As a result, copper is fairly restrictive in the CNS. Indeed, *C. neoformans* appears to sense copper limitation within the brain and upregulates expression of copper transporters, controlled by the copper-responsive transcription factor CUF1 [45]. The uptake of copper is important for the expression of several survival and virulence factors, including copper-dependent superoxide dismutases and melanin biosynthesis [45], and an inability to do so results in decreased dissemination to the brain [47]. For example, strains deficient in laccase, a copper-responsive enzyme needed for the production of melanin, have particularly poor dissemination rates to the brain [48], reinforcing that copper acquisition and subsequent expression of key virulence traits are required for the establishment of CM and progression of disease.

Interestingly, this response is specific to the brain microenvironment. In the lung, copper levels are far higher and *C. neoformans* instead faces a different challenge and needs to upregulate metallothioneins to counteract potential copper-mediated toxicity [45,49,50]. Whether copper restriction in the brain is a deliberate method of preventing infection or a self-preserving mechanism to prevent damage to neurons is not well understood. Further study into these mechanisms of ‘nutritional immunity’ is likely to yield promising new insights into how trace metals provide organ-specific immune responses to control infection, and may provide further clarity into how copper acquisition by *C. neoformans* could be targeted to prevent CM.

## 6. Brain Microvascular Endothelial Cells (BMECs)

The mechanisms governing *C. neoformans* crossing of the BBB, and particularly their interaction with BMECs that make up the initial layer of the BBB, represent an intense area of investigation within the field. Microbial invasion of the brain can occur via a variety of mechanisms. For example, some parasites produce proteolytic enzymes to cause physical disruption of the BBB [51], whereas receptor-mediated endocytosis following specific molecular interactions with BMECs is commonly seen with meningitis-causing bacteria [52]. The predominant mechanism used by *C. neoformans* to cross the BBB has been a matter of debate, with multiple groups putting forward evidence for an array of different routes (Figure 1). Paracellular migration across the BBB was shown to occur as a result of BMEC cytoskeleton remodeling and disruption of tight junctions, a process that is largely independent of the *C. neoformans* capsule, one of the most important virulence factors [53]. Direct uptake and subsequent transcellular migration of *C. neoformans* yeast by brain endothelial cells is also capsule-independent [54], requiring specific interactions between hyaluronic acid on the *C. neoformans* surface and CD44-containing lipid rafts expressed by BMECs to mediate fungal adhesion to these specialized cells of the BBB [54,55,56,57]. Other pathways may also operate independently to promote transcellular migration. For example, secretion of the Mpr1 metalloprotease was shown to mediate *C. neoformans* crossing of the BBB both in vitro and in vivo, and also permitted brain infection by non-pathogenic fungi engineered to express *MPR1* [58]. However, using intravital microscopy to visualize *C. neoformans* brain invasion in vivo in real time, this approach led to the observation that yeast cells in blood vessels became “stuck” after arriving at a vessel that was of a similar width to the yeast cells, with no evidence of *C. neoformans* adhering to BMECs prior to stopping [59], in line with previous reports of passive BBB crossing in vitro [54]. After this physical stopping mechanism, viable yeast cells were able to cross into the brain parenchyma using a mechanism that depended on urease expression by the fungus [59].

A further mechanism that has received much attention is the Trojan Horse route of brain infection. Several microbes and viruses have been described to enter host tissues as passengers within host cells, thus evading detection and clearance by the immune system. Early experiments showed that brain infection was higher in animals infected with *C. neoformans*-infected macrophages compared to animals infected with yeast cells alone [60], indicating that Trojan Horses might be a particular virulence trait of *C. neoformans* meningitis. More recently, Doering and colleagues employed a sophisticated in vitro system to accurately model the kinetics and efficiency of Trojan Horse traversal of the BBB by *C. neoformans*. By sorting macrophages infected with a single yeast cell, they were able to show that Trojan Horse phagocytes do cross the BBB via a transcellular route and contribute to dissemination, although not to the same degree as free yeast cells. However, only Trojan Horse phagocytes permitted infection of fungal mutants that are otherwise unable to penetrate the brain (e.g., urease-deficient and hyaluronic acid-deficient strains) [61]. Therefore, it appears that *C. neoformans* crosses the BBB using a range of different mechanisms, although many questions remain. For example, the influence of anatomical location within the brain on different transversal mechanisms is not yet known, and neither are the molecular responses of BMECs to *C. neoformans* interaction and how this impacts on infection outcome.

## 7. T-cells

While this review aims to focus on “resident” cells of the CNS, it is worthwhile mentioning that although lymphocytes (e.g., T-cells, B-cells and Natural Killer (NK) cells) are generally thought of as a recruited inflammatory cell, there is a growing body of evidence that some lymphocyte populations are involved in CNS immunity under homeostatic conditions. Although often thought of as immune-privileged, the CNS does have its own lymphatic system [62]. Lymphatic vessels absorb CSF from the sub-arachnoid space, which is found between the different layers that make up the meninges (Figure 1), and drain into deep cervical lymph nodes at the base of the skull. While extremely rare, T-cells are found here and are predominantly T_reg_ and memory effector phenotypes [63,64]. The role of these T-cells is not clear, but they may be involved in immune surveillance of the CNS.

Upon *C. neoformans* infection of the CNS, there is a massive recruitment of lymphocytes into the CSF, including CD4^+^ T-cells, CD8^+^ T-cells, B-cells, and NK cells [29]. CD4^+^ T-cells are thought to be the most important effector cell in the protection against CM [27], since adoptive transfers of CD4^+^ T-cells can help protect against CNS infection [65] and CD4^+^ T-cell deficiencies result in a profound susceptibility to CM in mice and humans. In particular, Th1-polarised CD4^+^ T-cells are thought to be protective since there is a strong induction of Th1-related cytokines in the brain of *C. neoformans*-infected mice, and a lack of these cytokines promotes susceptibility [26,66,67]. In addition to their essential role in controlling fungal proliferation within the brain, CD4^+^ T-cells are also required for the proper recruitment of other inflammatory cells into the brain, such as neutrophils and monocytes [20,68], as well as enhancing anti-fungal functions of microglia [26]. Interestingly, CD8^+^ T-cells are less able to promote fungal killing by microglia [26], which may help explain early studies that showed that CD8^+^ T-cells are not required for control of brain fungal infection [27].

Despite their clear protective roles in controlling CM, CD4^+^ T-cells can also be detrimental. A subset of HIV-infected patients who begin anti-retroviral therapy and exhibit improving CD4^+^ T-cell counts, can subsequently develop an inflammatory disorder associated with an opportunistic infection, termed ‘immune reconstitution inflammatory syndrome’ (IRIS) [69]. The paradoxical development of an infection upon regaining immune function is not well understood. Cryptococcal IRIS is thought to be the result of patients developing a new *C. neoformans* infection (relapsing) or the reactivation of a latent or sub-clinical infection following anti-viral therapy, which in turn activates pathologic CD4^+^ T-cell mediated immune responses to fungal antigens [3].

Indeed, recent data using mouse models of cryptococcal-IRIS indicated that CD4^+^ T-cells mediate both fungal clearance and immunopathology in the brain, and that depletion of CD4^+^ T-cells in this model could reverse neurological defects and reduce mortality in infected mice [20,70]. Therefore, underlying conditions of patients strongly influence the role of CD4^+^ T-cells in the pathogenesis of CM, and careful analysis of their protective and paradoxical functions will be needed prior to any development of therapies that modulate their behavior.

## 8. Other Lymphocytes

In addition to T-cells, B-cells have also been shown to restrict *C. neoformans* growth in the brain [71], and naturally-occurring antibodies are protective [72]. NK cells also play important protective roles in the defense against *C. neoformans* in the brain, in part by managing cryptococcomas, which are large tumor-like masses that can develop in the lung and brain. Cryptococcomas have acidic centers, which can limit the immune functions of many inflammatory cells. However, NK cells were shown to maintain their anti-cryptococcal functions at low pH and were found to infiltrate human cerebral cryptococcoma [73]. Interestingly, NK cells were recently observed to interact with neural stem cells in the brain following chronic inflammation, and this relationship helped shape subsequent tissue repair pathways [74]. Therefore, it will be interesting to determine not only the mechanisms that lymphocytes employ to control *C. neoformans* infection in the brain, but also the consequences of these host–pathogen interactions on downstream repair pathways and brain function.

## 9. Concluding Remarks

Human fungal diseases remain a significant global human health problem. There is an urgent need for better diagnostic tools and a wider array of therapies to treat these dangerous infections. An improved understanding of the mechanisms controlling immunity to these pathogens, particularly in an organ-specific context, will help reveal how fungi establish infections and potential immune pathways that may enable us to target their growth and promote recovery. *Cryptococcus* species are among the most significant causes of infectious meningitis in humans, yet there are many questions as to how the growth of these fungal pathogens is controlled within the CNS. For example, it is not yet known whether glia or resident macrophages are required for fungal clearance in the brain, or how they recognize and interact with invading yeast cells and recruit inflammatory effector immune cells. Answering these questions will help us to understand how this delicate organ protects itself against infection and provide new insights into how to treat these often devastating infections in vulnerable patients.

## Figures and Tables

**Table 1:** Distinguishing features of non-parenchymal macrophages and microglia.

	Turnover Rate	Surface Markers	Developmental Transcription Factors
Microglia	Long-lived	CD45^int^ CD11b Cx3CR1 Iba-1 Tmem119 MHCII^low^	PU.1 IRF8 CSF1R Sall1
Perivascular Macrophages	Long-lived	CD45^hi^ CD11b Cx3CR1 Iba-1 CD163 MHCII^hi^ CD206	PU.1 CSF1R
Choroid Plexus Macrophages	Partial turnover from monocytes	CD45^hi^ CD11b Cx3CR1 Iba-1 MHCII^hi^	PU.1 CSF1R
Meningeal Macrophages	Long-lived	CD45^hi^ CD11b Cx3CR1 Iba-1 MHCII^hi^ CD206	PU.1 IRF8 CSF1R

^int^: intermediate expression; ^hi^: high expression; ^low^: low expression.
